# 5-Fluorouracil Treatment Alters the Efficiency of Translational Recoding

**DOI:** 10.3390/genes8110295

**Published:** 2017-10-31

**Authors:** Junhui Ge, John Karijolich, Yingzhen Zhai, Jianming Zheng, Yi-Tao Yu

**Affiliations:** 1 Department of Pathology, Changzheng Hospital, Second Military Medical University, Shanghai 200003, China; gejunhui@akmpath.com (J.G.); zhaiyingzhen@akmpath.com (Y.Z.); 2 Department of Pathology, Microbiology, and Immunology, Vanderbilt University Medical Center, Nashville, TN 37232-2363, USA; john.karijolich@vanderbilt.edu; 3 Department of Pathology, ChangHai Hospital, Second Military Medical University, Shanghai 200433, China; 4 Department of Biochemistry and Biophysics, University of Rochester School of Medicine and Dentistry, Rochester, NY 14642, USA

**Keywords:** 5-Fluorouracil, translational recoding, internal ribosome entry site, programmed frameshifting, nonsense suppression, RNA pseudouridylation

## Abstract

5-fluorouracil (5-FU) is a chemotherapeutic agent that has been extensively studied since its initial development in the 1950s. It has been suggested that the mechanism of action of 5-FU involves both DNA- and RNA-directed processes, but this has remained controversial. In this study, using a series of in vivo reporter constructs capable of measuring translational recoding, we demonstrate that cells exposed to 5-FU display a reduced capacity to engage in a variety of translational recoding events, including +1 programmed frameshifting (PRF) and −1 PRF. In addition, 5-FU-treated cells are much less accurate at stop codon recognition, resulting in a significant increase in stop codon-readthrough. Remarkably, while the efficiency of cap-dependent translation appears to be unaffected by 5-FU, 5-FU-treated cells display a decreased ability to initiate cap-independent translation. We further show that knockdown of thymidylate synthase, an enzyme believed to be at the center of 5-FU-induced DNA damage, has no effect on the observed alterations in translational recoding. On the other hand, ribosomal RNA (rRNA) pseudouridylation, which plays an important role in translational recoding, is significantly inhibited. Taken together, our results suggest that the observed effect of 5-FU on recoding is an RNA-directed effect. Our results are the first to show definitely and quantitatively that translational recoding is affected by exposure to 5-FU. Thus, it is possible that a substantial portion of 5-FU cytotoxicity might possibly be the result of alterations in translational recoding efficiency.

## 1. Introduction

Fluoropyrimidine 5-fluorouracil (5-FU) was initially developed in the 1950’s following the observation that rat hepatomas metabolized uracil more rapidly than other nucleotides, implicating uracil metabolism as a potential antimetabolic target [1]. Today, 5-FU is commonly used in the treatment of a variety of solid tumors such as colorectal, breast, and liver carcinomas [2]. Although nearly six decades have passed since the initial uses of 5-FU as a chemotherapeutic agent, its mechanism of action remains an issue of debate.

5-FU, which enters cells through the same facilitated transport mechanism used by uracil, is intracellularly converted into several active metabolites, including fluorodeoxyuridine monophosphate (FdUMP), fluorodeoxyuridine triphosphate (FdUTP), and fluorouridine triphosphate (FUTP) [3,4]. The cytotoxicity associated with these metabolites was initially ascribed to their inhibition of the enzyme thymidylate synthase (TS). TS is responsible for the reductive methylation of deoxyuridine monophosphate (dUMP) to deoxythymidine monophosphate (dTMP), and is the sole source of thymidylate intracellularly [5,6]. FdUMP forms stable interactions with TS, resulting in accumulation of dUMP/dUTP and a simultaneous reduction in the levels of deoxythymidine triphosphate (dTTP) [7,8,9,10,11]. The reduction of dTTP potentiates the incorporation of dUTP into DNA, resulting in DNA damage. Repair of uracil and 5-FU-containing DNA requires removal of the misincorporated nucleotide by the nucleotide excision repair enzyme uracil-DNA-glycosylase (UDG) and repair of the lesion by DNA polymerase and DNA ligase [12]. Paradoxically, however, treatment of 5-FU-exposed cells with thymine does not negate the cytotoxic effects associated with 5-FU exposure, arguing against TS inhibition (a DNA-directed or DNA-based process) as the main determinant of 5-FU cytotoxicity.

Given that 5-FU can be readily converted into 5-fluorouridine triphosphate (5-FUTP), a ribonucleotide analog that can be incorporated into RNAs, it has been proposed that 5-FU may directly affect various aspects of gene regulation mediated by RNA, including pre-messenger RNA (mRNA) splicing, pre-ribosomal RNA (rRNA) processing and protein synthesis [13,14,15,16]. Indeed, significant correlations exist between 5-FU incorporation into RNA and the loss of clonogenic potential in human colon and breast cancer cell lines [17,18]. Furthermore, experimental data has directly demonstrated that 5-FU treatment results in defects in the processing of rRNA and pre-mRNA splicing in various systems [19,20,21,22,23,24,25], likely due to inhibition of naturally-occurring RNA pseudouridylation by 5-FU (5-FU is considered a potent pseudouridylation inhibitor) [24] and/or other 5-FU-induced, RNA-directed effects. In addition, wild type *Saccharomyces cerevisiae* and mutants lacking nonessential transfer RNA (tRNA) modification enzymes are temperature sensitive and hypersensitive to 5-FU, respectively [26]. 

5-FU incorporation into RNA has also been suggested to stimulate miscoding of mRNA in *Escherichia coli*, as 5-FU treatment resulted in the synthesis of an inactive β-galactosidase enzyme [27]. In addition, exposure to 5-FU resulted in the phenotypic reversal of an amber alkaline phosphatase mutant of *E. coli*, presumably by mispairing with cytosine [28]. However, whether 5-FU treatment of mammalian cells results in a reduction in translational accuracy has yet to be extensively investigated. In this regard, translational accuracy has been primarily analyzed in vitro using either mRNAs purified from 5-FU treated cells or with in vitro transcribed mRNAs that are fully substituted with 5-FU. In these experimental systems, 5-FU did not induce any alterations in mRNA decoding [29,30,31]. In addition, when in vitro transcribed TS mRNA, which is 100% substituted with 5-FU, was translated in rabbit reticulocyte lysate, fully functional TS was generated, and found to be indistinguishable from TS generated from an in vitro transcript synthesized with uridine [32]. While these studies would suggest that translational accuracy is not affected in 5-FU treated mammalian cells, this relies on the assumption that only the mRNA template is responsible for accurate mRNA decoding. However, this assumption is incorrect as alterations in rRNA, tRNA, as well as mRNA, can affect the decoding of mRNA. Thus, the best way to accurately determine the effects of 5-FU treatment of mammalian cells on translation accuracy is to measure translation in vivo. 

In the present study we address the role of 5-FU treatment on translational accuracy using a purely in vivo system, which allows us to quantitatively measure translational recoding. We show that while 5-FU treatment did not affect the efficiency of cap-dependent translation, significant changes in the efficiency of various translational recoding events, including programmed frameshifting, nonsense suppression or stop-codon readthrough, and cap-independent translation, were observed. Furthermore, we provide evidence that 5-FU-induced alterations in translational recoding involve an RNA (rather than DNA)-directed process (likely through inhibition of rRNA pseudouridylation). Therefore, our results provide novel insights into the mechanism of 5-FU cytotoxicity. 

## 2. Materials and Methods 

### 2.1. Genetic Methods and Plasmid Construction

To construct the dual-luciferase reporter series plasmids, the dual-luciferase coding region including recoding element, was removed by digestion with SpeI and XhoI from pJD375, 431, 432, 433 [33], and inserted into NheI and XhoI digested and purified pcDNA 3.1 (Invitrogen, Carlsbad, CA, USA), creating pcDNA-control (pcDNA-2luci), stop codon UAA (pcDNA-UAA), stop codon UAG (pcDNA-UAG), stop codon UGA (pcDNA-UGA). For constructs containing the recoding sequences of human immunodeficiency virus type1 (HIV-1) gag-pol frameshift (pcDNA-HIV), human ornithine decarboxylase antizyme (pcDNA-OAZ), and hepatitis C virus (HCV) internal ribosome entry site (IRES) element (pcDNA-IRES), complementary oligonucleotides with Eco47III and BamHI compatible ends were synthesized. Then they were used to generate HIV and OAZ frameshifting sequences, respectively, via polymerase chain reaction (PCR). Restriction-digested PCR products were cloned between the unique Eco47III and BamHI sites of pcDNA-2luci.

### 2.2. Cells and Cell Culture

SW-480 human cells used in this study were obtained from the American Tissue Type Culture Collection (Manassas, VA, USA) and were maintained in Dulbecco’s modified Eagles medium supplemented with 10% fetal bovine serum (HyClone, Logan, UT, USA). All transfections were performed using lipofectamine 2000 (Invitrogen) according to the manufacturer’s instructions. 5-FU or uracil was added to medium 6 h after transfection.

### 2.3. Dual-Luciferase Assays

Luciferase activity in SW-480 cells was determined 30 h after transfection. Following removal of the growth medium cells were washed once with phosphate-buffered saline (PBS). 200 μL of passive Lysis Buffer reagent (Promega, Madison, WI, USA) was then added directly to the culture well. Concentrations of crude lysates were adjusted to the same protein concentration, as determined by the Bradford Protein Assay (BioRad, Hercules, CA, USA) according to manufacturer’s instructions, and fifty microliters of lysate was assayed by the Dual-Luciferase reporter assay (Promega) and a TD 20/20 luminometer (Turner Designs, San Jose, CA, USA), according to manufacturer’s instructions.

Frameshift efficiencies were calculated using the method described previously [34]. The firefly/*Renilla* activity ratio generated from the control reporter (pcDNA375) was divided by that from the frameshift reporters, stop codon and HCV IRES and multiplied by 100% to obtain frameshift efficiencies for each recoding signal. All assays were performed at least three times. To determine the linearity of the assay, 1:5 serial dilutions of lysates from cells harboring the pcDNA-control and pcDNA 376 were prepared in lysis buffer and luciferase activities were determined as described above.

### 2.4. Real-Time Reverse Transcriptase -PCR for Detecting Dual Luciferase mRNA

Two micrograms of total RNA were treated with DNase (Invitrogen) and 1 μg was used for complementary DNA (cDNA) synthesis. The reaction was carried out using random decamers and the First strand cDNA synthesis kit (Invitrogen) following the manufacturer’s recommendations. 

PCR primers used were as follows: forward 5′-AAGCCATACCAAACGACGAG-3′, reverse 5′-TTGCCGGGAAGCTAGAGTAA-3′. β2-Microglobulin was used as a reference for normalization Forward 5′-CGGCAGGCATACTCATCTTT-3′, Reverse: 5′-GGTTTCATCCATCCGACATT-3′, and relative quantification was analyzed using iCycler iQ Optical System Software Version 3.0a (BioRad). Real-time PCR was performed using a Bio-Rad iQ iCycler Detection System (Bio-Rad) with SYBR green fluorophore (Bio-Rad). Reactions were performed in a total volume of 20 μL-including 10 μL 2× SYBR Green PCR Master Mix (Applied Biosystems, Foster City, CA, USA), 5 μL of each primer at 5 μM concentration, and 1 μL of the previously reverse-transcribed cDNA template. Protocols for each primer set were optimized using five serial 10× dilutions of template cDNA obtained from SW-480. PCR reactions consisted of 30 cycles with optimal conditions as follows: 94 °C for 20 s; 50 °C for 1 min; 72 °C for 30 s; and an optimized collection data step, 80 °C for 5 s. All samples were run in triplicate. 

### 2.5. siRNAs and Transfection

All small interfering double-stranded RNA (siRNAs) were obtained from Dharmacon RNA Technologies (Lafayette, CO, USA) as annealed and desalted duplexes. ON-TARGET plus or siGENOME reagents were used as recommended by Dharmacon RNA Technologies for each targeted mRNA sequence. The TS siRNA targets nucleotides (nts) 526–544 using sequence 5′-GGACUUGGGCCCAGUUUAU-3′. Nontargeting control siRNA was used as a control. All siRNAs were transfected using Lipofectamine 2000 reagent (Invitrogen) according to manufactures instructions. 

### 2.6. Western Immunoblot Analysis

SW-480 cells were plated, transfected, and harvested. Cell pellets were resuspended in cell lysis buffer containing freshly added phenylmethylsulfonyl fluoride protease inhibitor (Thermo Fisher Scientific, Waltham, MA, USA). Equivalent amounts of protein (30 μg) from each cell lysate were resolved on sodium dodecyl sulfate polyacrylamide gel electrophoresis (SDS-PAGE). Gels were electroblotted onto nitrocellulose membranes. Membranes were incubated for 1 h with primary antibodies at the following dilutions: Anti-Thymidylate Synthase monoclonal antibody, 1:100; anti-α-tubulin monoclonal antibody, 1:5000 (Abcam, Cambridge, UK). After five 10-min washes in 1× PBS with 0.1% Tween-20, membranes were incubated with a dilution of 1:100 of horseradish peroxidase-conjugated secondary antibody (IgG goat antimouse; Abcam) for 1 h at room temperature. After an additional five 10-min 1× PBS with 0.1% Tween-20 washes, membranes were processed by the enhanced chemiluminescence method (SuperSignal West Pico substrate; Pierce, Rockford, IL, USA), and protein bands were visualized by autoradiography.

### 2.7. [^32^P]-Orthophosphate Labeling of rRNA in SW-480 Cells

SW-480 cells were grown in 100-mm culture dishes to near confluence. One day before labeling, cells were incubated with 5-FU or uracil. One hour before labeling, growth media were removed and replaced with prewarmed phosphate-free DMEM containing 5-FU or uracil. [^32^P]-orthophosphate (NEN Life Science Products, Boston, MA, USA) was added to the medium to a final concentration of 20 μCi/mL and incubated for 24 h at a 37 °C in a CO_2_ humidified cell culture incubator. The incubations were terminated by removing medium, and the cells were washed twice with ice-cold PBS to remove residual radioactive phosphate. Total RNA was purified using the TRIzol reagent (Invitrogen) following the manufacturer’s recommendation.

### 2.8. Two-Dimensional-Thin Layer Chromatography Pseudouridylation Assay (2D-TLC)

Total RNA was resolved in 3-(*N*-morpholino) propanesulfonic acid (MOPS)/formaldehyde agarose gels containing 2.2 M formaldehyde both in the gel and in the 1× MOPS running buffer, essentially as described in [1]. The voltage gradient was 5 V/cm. Then 18S and 28S rRNA were recovered from the agarose gel by elution in to dialysis tubing. The isolated rRNA was phenol-chloroform extracted and precipitated with 100% ethanol. The RNA pellet was resuspended in 10 μL of 50 mM NH_4_ acetate (pH 5.3) containing 0.2 μg of nuclease P1 to (MP Biomedicals, cat. No. 195352, Solon, OH, USA). 3 µL of the digestion reactions were spotted on to cellulose thin layer chromatography (TLC) plates (Cellulose CEL 400-10, 20 × 20 cm; Macherey-Nagel, Düren, Germany). The first dimension was run in isobutyric acid/water/ammonia (66:33:1, *v*/*v*/*v*). The plate was dried in air and developed in the second dimension with isopropanol/concentrated HCl/water (70:15:15, *v*/*v*/*v*). The plate was dried and the radioactivity analyzed by phosphorimager analysis (Typhoon FLA 9500, GE Healthcare, Pittsburgh, PA, USA).

### 2.9. Analysis of rRNA and U2 snRNA Levels

Total RNA was resolved by electrophoresis on 0.8% agarose gel containing 2.2 M formaldehyde both in the gel and in the 1× MOPS running buffer for 3–4 h at 25 V. After electrophoresis, gels were photographed. To analyze the levels of U2 (as a loading control), gels were blotted overnight onto nylon membranes by capillary action. Membranes were recovered by rinsing in 2× saline-sodium citrate buffer (SSC), cross-linked using a ultra violet (UV) Stratalinker (Stratagene, La Jolla, CA, USA). U2 small nuclear RNA (snRNA)was detected by standard northern blotting techniques. The membrane was hybridized with 20 pmol of [^32^P]-labeled antisense U2 probe (complementary to nucleotides 25–45) in 50 mL of hybridization buffer (6× SSC (from a 20× stock of 3 M NaCl, 0.3 M. Sodium Citrate, pH 7.0), 5× Denhardt’s solution (from a 50× stock of 1% Ficoll, 1% BSA, 1% polyvinylpyrrolidone), 25 mM sodium phosphate buffer, pH 6.5, 0.5% SDS) at 37 °C for 1 h or longer using 30 cm hybridization bottles and a hybridization oven (Hogentogler & Co., Inc., Columbia, MD, USA). The membrane was then washed twice with 30 mL of 1× SSC, 0.1% SDS at 37 °C for 15 min and analyzed by phosphorimaging.

## 3. Results

### 3.1. 5-FU Treated Cells Have Defects in Translational Recoding

As 5-FU-based treatment regimes are currently the gold standard for colon cancer, we used SW-480 cells (colon cancer cell line) to investigate the effects of 5-FU treatment on translational recoding. In order to assess translational competency in 5-FU-treated cells, we constructed a series of dual-luciferase reporters to measure various modes of translational recoding. We chose to use a bicistronic reporter system as they are internally controlled, eliminating the need for indirect methods of normalization and controlling for changes that may arise from differential mRNA abundance when using separate reporters. 

**Figure 1:**
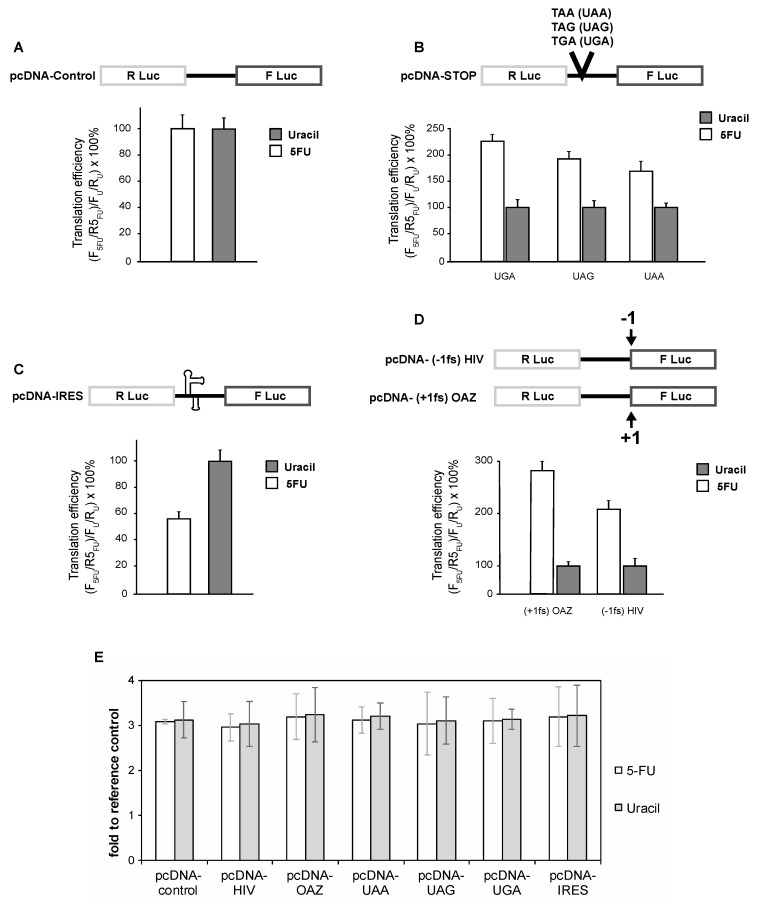
The efficiency of translational recoding is altered by 5-fluorouracil (5-FU) treatment. SW-480 cells harboring the indicated luciferase reporter plasmids were treated with 10 μM 5-FU or 10 μM uracil for 24 h before determining luminescence (**A**–**D**). qRT-PCR analysis of reporter mRNA indicates that 5-FU treatment does not affect reporter mRNA levels (**E**). HIV: human immunodeficiency virus; OAZ: ornithine decarboxylase antizyme; IRES: internal ribosome entry site.

To assess cap-dependent translation we transfected SW-480 cells with a dual-luciferase reporter construct. Six hours following transfection, cells were treated with 10 μM 5-FU or 10 μM uracil for 24 h. At this concentration (10 μM 5-FU), the cells grew healthy for at least 4 days. Figure 1A shows that both 5-FU- and uracil-treated cells initiate cap-dependent translation with a similar efficiency. Analysis of the steady state levels of the reporter mRNAs by real-time reverse transcriptase (RT)-PCR indicated that the reporter mRNAs were present at similar levels (Figure 1E). 

Next, we constructed dual-luciferase constructs to assess both +1 and −1 ribosome programmed ribosome frameshifting (PRF). To determine the effects of 5-FU treatment on −1 frameshifting, the −1 frameshift signal of the human immunodeficiency virus type1 (HIV-1) gag-pol frameshift was inserted between *Rennilla* luciferase (RLuc) and firefly luciferase (FLuc), resulting in the firefly luciferase gene being in the −1 reading frame (Figure 1B). Cells treated with 5-FU had a significant enhancement in the ability to engage in −1 frameshifting (Figure 1B). Specifically, there was a 115% enhancement in −1 PRF. To assess +1 PRF we inserted the human ornithine decarboxylase antizyme (pcDNA-OAZ) +1 frameshift signal between RLuc and FLuc such that the FLuc gene was in the +1 reading frame. Similar to −1 PRF, the ability to engage in +1 PRF was significantly enhanced in 5-FU-treated cells compared to uracil-treated cells (Figure 1B). Analyses of both the −1 and +1 PRF reporter transcripts demonstrated that there were no sequence changes and that all mRNA transcripts were present at a similar level (Figure 1E). 

To determine the effects of 5-FU treatment on translation termination we constructed three dual-luciferase reporters, each of which harbored one of the three termination codons at the sixth codon position of the FLuc gene (Figure 1C). Interestingly, cells which were treated with 10 μM 5-FU were hypoaccurate in recognizing termination codons relative to cells treated with 10 μM uracil (Figure 1C). Specifically, 5-FU treated cells were 124%, 82%, and 45% less accurate at recognizing UGA, UAG, and UAA termination codons, respectively (Figure 1C). Furthermore, there were no changes in reporter sequences, and all reporter mRNAs were present in similar quantities (Figure 1E). 

While the bulk of cellular translation is initiated in a cap-dependent manner, an increasing number of transcripts are being identified that can initiate translation in a cap-independent, or IRES dependent, manner. Importantly, IRES elements have been identified in a number of genes implicated in malignant transformation [35]. Furthermore, IRES-dependent translation is used by a number of clinically significant viruses [36]. To determine the effects of 5-FU treatment on IRES-dependent translation, we transfected SW-480 cells with a previously characterized dual-luciferase construct containing the HCV IRES between RLuc and FLuc. Analysis of HCV activity indicated that 5-FU treated cells had a 43% decrease in internally initiated translation at the HCV IRES (Figure 1D). For both 5-FU and uracil-treated cells, reporter mRNA sequence remained unchanged and reporter mRNA transcript levels were similar. (Figure 1E). 

### 3.2. The Effect of 5-FU on Translational Recoding Does Not Involve DNA-Directed Process

**Figure 2:**
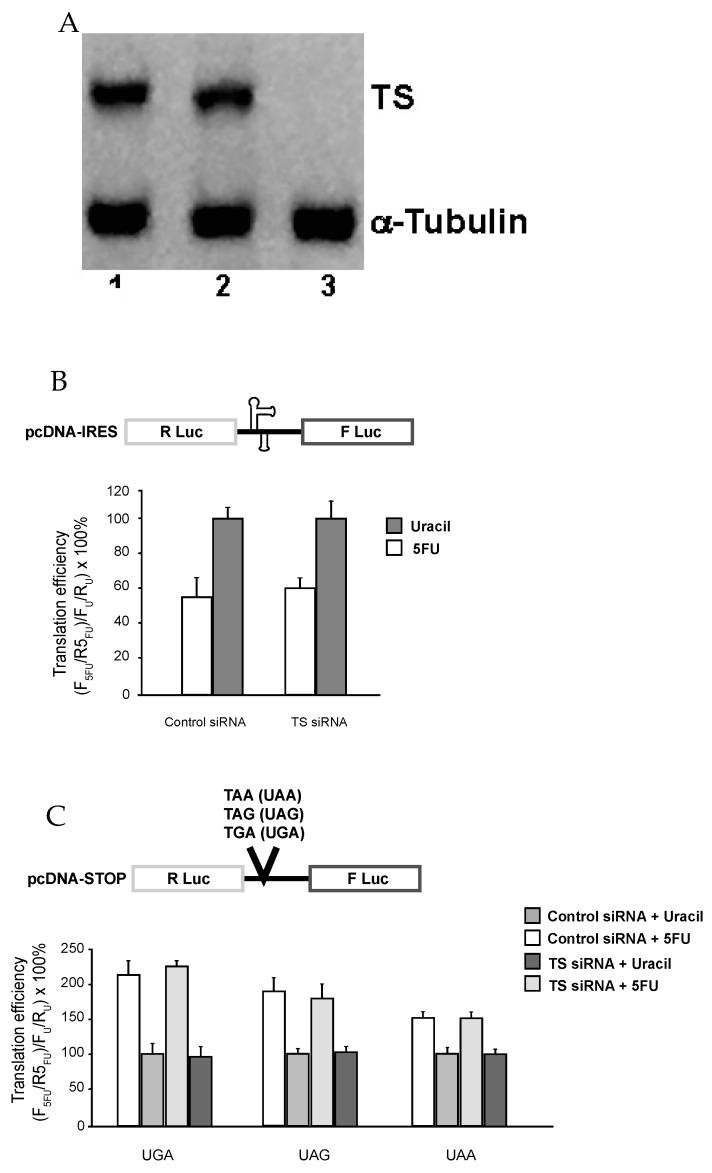
Effects of 5-FU on translation recoding are independent of thymidylate synthase (TS) activity. (**A**) SW-480 Cells were incubated in the absence (Lane 2) or presence (Lanes 3) of TS-targeted small interfering double-stranded RNA (siRNA) (1 nm)-Oligofectamine complexes for 48 h and then harvested and processed for Western blot analysis as described in “Materials and Methods”. Lane 1 corresponds to treatment with Oligofectamine complexes containing 1 nm Control siRNA. (**B**) and (**C**) SW-480 cells harboring the indicated luciferase reporter plasmids were treated with either control- or TS-siRNA for 48 h prior to being treated with 10 μM 5-FU or 10 μM uracil for 24 h before determining luminescence.

Next, we decided to test whether the observed effect of 5-FU on translational recoding involves DNA-directed process. In this regard, it has been reported that 5-FU, upon being converted into FdUMP, binds to TS and blocks its function in converting dUMP into dTMP (which will normally be further converted into dTTP). Consequently, dUMP/dUTP accumulated, whereas the level of dTMP/dTTP was drastically reduced, thus increasing the possibility of incorporation of dUTP into DNA (DNA damage). Clearly, TS is a determinant in this DNA-based process. To test whether this process is involved, we knocked down TS expression using siRNA prior to 5-FU treatment. As seen in Figure 2A (Western blot), TS siRNA, which targets nt 526–544, almost completely inhibited the expression of TS protein (lane 3). In contrast, treatment with no siRNA (lane 2) or a control siRNA (lane 1) had no effect on TS level. We also measured the expression of a control protein, α-tubulin, and found that its level remains unchanged in all three samples (lanes 1–3). 

TS-intact and TS-knockdown cells were treated with 5-FU, and translational recoding was measured. As shown in Figure 2B,C, 5-FU induced alterations in recoding, including cap-independent translation (Figure 2B) and stop-codon read-through (Figure 2C), were unaffected, suggesting that TS (and likely DNA-based process) is not involved.

### 3.3. 5-FU Inhibits rRNA Pseudouridylation

Since the observed 5-FU-induced alterations in recoding did not seem to be a DNA-based process, we turned our attention to rRNA and its modification. It has been demonstrated that loss of rRNA pseudouridylation results in an impairment of ribosome assembly, translation and translational recoding [37,38,39,40]. In addition, 5-FU is widely used as a pseudouridylation-specific inhibitor. To further explore the molecular details behind the alterations in decoding by 5-FU, we decided to carry out the pseudouridylation assay to measure the level of rRNA pseudouridylation. 

**Figure 3:**
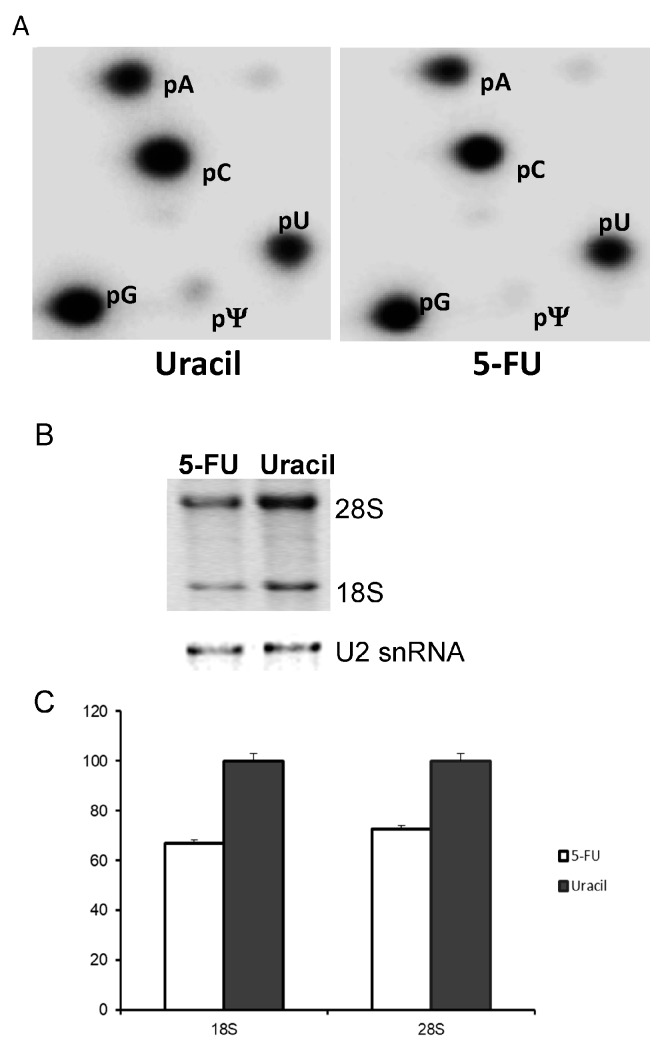
Ribosomal RNA (rRNA) pseudouridine levels as well as rRNA levels in SW-480 cells treated with uracil or 5-FU. (**A**) 18S and 28S rRNA were gel-purified from [^32^P]-orthophosphate pulsed SW-480 cells cultured in medium containing uracil (left panel) or 5-FU (right panel), and subjected to nuclease P1 digestion and 2-dimensional thin layer chromatography (2D-TLC). Spots corresponding to adenosine (pA), cytosine (pC), guanosine (pG), uridine (pU), and pseudouridine (pΨ) are labeled. For uracil-treated cells (left panel), the pU/pΨ ratio is ~5%; for 5-FU-treated cell (right), the pU/pΨ ratio is ~1%. (**B**) Total RNA from 5-FU- and uracil-treated cells was resolved by electrophoresis, and photographed (top). As a loading control, U2 small nuclear RNA (snRNA) was detected by Northern blotting (bottom). (**C**) Relative levels of 18S and 28S from 5-FU- and uracil-treated cells were quantified.

In order to quantitatively determine pseudouridine (Ψ) levels, SW-480 cells were treated for 24 h with either 10 μM 5-FU or 10 μM uracil. One day prior to harvesting cells, [^32^P]-orthophosphate was added to the medium to a final concentration of 20 μCi/mL. rRNA was purified and subjected to nuclease P1 cleavage and 2D TLC (Figure 3A). We detected pseudouridylate-5′-monophosphate (pΨ) in both uracil-treated (left panel) and 5-FU-treated (right panel) cells. However, quantification of the pΨ/pU ratio indicates there is approximately a 5-fold reduction in Ψ in the 5-FU treated cells. Although we were unable to directly visualize the 5-F-uridylate-5′-monophosphate spot by 2D TLC (no additional pFU spot was detected in TLC of 5-FU-treated cells as compared to TLC of uracil-treated cells), it is possible that 5-FU is incorporated into rRNA at a level below the detection limit. Yet, such a low level of incorporation is nonetheless still sufficient to reduce rRNA pseudouridylation (through binding and sequestration of pseudouridylation enzymes). In this regard, it has been reported that culturing cells in medium containing 5-FU allows a low level incorporation of 5-FU into U2 snRNA, resulting in specific inhibition of U2 pseudouridylation [24]. In addition, we also detected a small reduction of steady-state level of rRNA in 5-FU treated cells compared to uracil treated cells (Figure 3B,C). Thus, although additional studies are necessary to fully evaluate the effect of 5-FU on rRNA stability, our results nonetheless suggest that 5-FU-containing rRNA would be the likely target of the decay machinery as it is most likely nonfunctional and may even form adducts with the modification enzymes. We also performed in vitro translation experiments using 5-FU-incorporated mRNA, but failed to detect any significant alterations in recoding (data not shown). 

Taken together, these results are consistent with the notion that 5-FU, when included in the medium, is able to inhibit rRNA pseudouridylation, resulting in alterations in translational recoding.

## 4. Discussion

The mechanism of 5-FU cytotoxicity has been in debate since its initial development as a chemotherapeutic agent. Previous studies have shown that 5-FU treatment alters several RNA-mediated processes, including snRNA pseudouridylation, pre-mRNA splicing [24] and rRNA biogenesis [13,19]. However, to date, the effects of 5-FU treatment on the maintenance of translational fidelity were not well documented. The only in vivo evidence for altered translation upon 5-FU treatment came from *E. coli* [41]. Here, we present evidence that 5-FU treatment severely alters translational recoding in mammalian cells, in particular, +1 and −1 programmed frameshifting, nonsense suppression, and cap-independent translation. Interestingly, the ability to properly engage in cap-dependent translation remained unaffected. In addition, we show that 5-FU is capable of inhibiting rRNA pseudouridylation, a modification that is known to be important for translational recoding [40]. Our results therefore argue that at least a portion of 5-FU cytotoxicity can be attributed to inhibition of rRNA pseudouridylation and alterations in translational recoding efficiency. It should be noted that the experiments presented here were carried out in SW480 cells (which model colon cancer), and that the extent of RNA- versus DNA-mediated effects of 5-FU may vary between cell lines. 

It is known that 5-FU, when included in medium, can be incorporated into various types of RNA. However, given that rRNA plays an important role in translation and recoding, we believe that the defects in recoding observed are at least partially the result of the incorporation of 5-FU into rRNA. Although it is possible that 5-FU, a modified nucleotide analog that is distinct from any other known nucleotides, affects RNA-RNA and/or RNA-protein interactions, thus directly contributing to translational recoding, for at least three reasons, we argue that it is more likely the subsequent inhibition of rRNA pseudouridylation that exerts an indirect effect on translational recoding. First, this argument is consistent with numerous reports indicating RNA pseudouridylation is critical to a number of cellular processes, including pre-mRNA splicing and protein translation [37,38,39,40,42,43]. Second, our results have shown that only an extremely low level of 5-FU (beyond detection) is incorporated into rRNA (Figure 3A), or in other words, there is only a very low level of 5-FU-containing rRNA present in the cell. On the other hand, we did observe a significant (~5-fold) decrease in rRNA pseudouridylation (Figure 3A). Third, our argument is also consistent with the earlier results of the failure to observe any defects in translation when only mRNA was substituted with 5-FU [29,30,31,32]. 

Regarding the molecular mechanism of how RNA pseudouridylation is inhibited, it has been reported that 5-FU, when included in the medium, can incorporate into U2 (a spliceosomal snRNA) at naturally occurring pseudouridylation sites. The 5-FU-incorporated U2 can in turn block pseudouridylation of newly synthesized U2 snRNA in a site-specific manner. This is achieved through tight binding between the 5-FU-incorporated U2 and the U2-specific pseudouridylases, and hence sequestration of U2-specific pseudouridylases that would otherwise modify U2 under normal conditions [24,42,44]. We believe that this mechanism also governs 5-FU-induced inhibition of rRNA pseudouridylation observed in the current work. However, more work is necessary to verify this speculation.

Malignant transformation of cells is a coordinated multi-step process requiring the misregulation of several genes and gene products. While the extent to which oncogenes and tumor suppressors engage in translational recoding is only beginning to emerge, whether the cytotoxicity of 5-FU is the result of a particular misregulation is uncertain and more work is necessary. 

## 5. Conclusions

Culturing cells in medium containing 5-FU results in reduction in rRNA pseudouridylation and alterations in translational recoding, which may contribute at least in part to 5-FU cytotoxicity.
